# Large-scale sampling of potential breeding sites in male ruffs

**DOI:** 10.1098/rspb.2024.2225

**Published:** 2025-01-08

**Authors:** Bart Kempenaers, Mihai Valcu, Theunis Piersma, Peter Santema, Raf Vervoort

**Affiliations:** ^1^ Department of Ornithology, Max Planck Institute for Biological Intelligence, Eberhard-Gwinner Strasse, Seewiesen 82319, Germany; ^2^ Rudi Drent Chair in Global Flyway Ecology, Groningen Institute for Evolutionary Life Sciences (GELIFES), University of Groningen, PO Box 11103, Groningen, 9700 CC, The Netherlands; ^3^ BirdEyes, Centre for Global Ecological Change at the Faculties of Science & Engineering and Campus Fryslân, University of Groningen, Zaailand 110, Leeuwarden 8911 BN, The Netherlands; ^4^ Department of Coastal Systems, NIOZ Royal Netherlands Institute for Sea Research, PO Box 59, Den Burg, Texel 1790 AB, The Netherlands

**Keywords:** nomadism, *Calidris pugnax*, site sampling, lek, polygyny, female-only care

## Abstract

The traditional narrative of the life cycle of migratory birds is that individuals perform long-distance movements between a breeding and a wintering site, but are largely resident at those sites. Although this pattern may apply to socially monogamous species with biparental care, in polygamous systems, the sex that only provides gametes may benefit from continuing to move and sample several potential breeding sites during a single breeding season. Such behaviour would blur the distinction between migration and breeding. We used satellite telemetry to study movements during the breeding season of males of the ruff *Calidris pugnax*, a lekking wader with a polygynous mating system and female-only parental care. Ruffs have a unique life-history, with three distinct genetically determined male mating strategies: aggressive ‘independents’, submissive ‘satellites’, and female-mimicking ‘faeders’. Within the breeding season, ruff males visited up to 23 sites (median: 11) and travelled up to 9029 km (median: 4435 km) covering a considerable part of their known breeding range. All three male morphs displayed breeding site sampling, indicating that they might gain similar benefits from this behaviour. Our findings suggest that large-scale breeding range sampling may be a common feature of migratory species with female-only care and strong male-male competition.

## 1. Introduction

Twice a year, migratory birds move between an area where they spend the non-breeding season and a site where they reproduce [1,2]. The traditional narrative of the annual cycle of migratory birds is that when individuals arrive at the breeding site, they stay there for the duration of the breeding season, after which they make a similar journey back to their wintering grounds [3]. Arrival at and departure from the breeding site thus marks the transition from a migratory phase to a sedentary phase and *vice versa*. However, most of our knowledge of the seasonal movements of birds comes from socially monogamous species with biparental care. The movements of polygynous migratory species are much less understood, in part because they typically have low site-fidelity [4], making it difficult to monitor them across seasons.

A study using satellite telemetry to track polygynous pectoral sandpipers *Calidris melanotos* revealed a lifestyle that deviates markedly from that of monogamous birds with biparental care [5]. The mating system of pectoral sandpipers is characterized by female-only care and intense competition between males for access to fertile females [6]. Most males that arrived at a particular breeding site stayed for a few days only. They then continued to travel, visiting a median of 10 other potential breeding sites (range: 1–23) across a large part of their breeding range, and covering on average more than 3000 km within a single breeding season [5]. Only 13% of males stayed at a single breeding site for the entire breeding season. The duration of a male’s stay at a site correlated strongly with the number of locally breeding females [5], suggesting that males sampled distant breeding sites to maximally exploit the spatial and temporal variation in the availability of fertile females. Large-scale sampling of potential breeding sites could be a unique feature of this particular species, or it could be a more general pattern in non-monogamous, migratory species.

Kempenaers & Valcu [5] hypothesized that large-scale sampling of potential breeding sites might occur in other migratory species with male-dominance polygyny. When males provide no offspring care, competition between males for access to females is high, and males cannot monopolize fertile females, it may be advantageous for males to look for mating opportunities over larger areas. Assuming that there is spatial variation in the timing of female fertility within a season, or that fertile females are available at a given site over a long enough period, males may increase their reproductive success by moving to another breeding site when they are locally unsuccessful, or when local mating opportunities are low or decreasing. This is especially true when temporal and spatial variation in the availability of fertile females is high, and for males unpredictable, between years. However, it remains unknown whether the large-scale breeding site sampling that was reported in pectoral sandpipers is a life-history trait unique to that species, or whether it occurs in other species where males do not provide parental care and are under strong sexual selection.

The ruff *Calidris pugnax* is a lekking wader (suborder Charadrii) with a polygynous mating system and female-only parental care [7,8]. Most of the population winters in Africa and breeds in Eurasia across a large latitudinal and longitudinal range encompassing temperate zones as well as subarctic and arctic regions [9,10]. Ruffs are unique among birds in that the population consists of three genetically determined morphs [11–13]. Among males, each morph has a distinct mating strategy and is markedly different in morphology and behaviour [7,14]. ‘Independents’ (80–90% of males) are aggressive, often (but not always) defend a small territory on a lek, and tend to have dark-coloured nuptial plumage [7]. ‘Satellites’ (10–20% of males) do not hold a territory, behave submissively towards and associate with independents, and tend to have mostly white nuptial plumage [7,14]. ‘Faeders’ (approx. 1% of males) resemble females in their plumage and do not display male-typical courtship behaviours, but presumably attempt to sneak copulations with females that visit a lek [15,16].

Previous observations provide indirect evidence that male ruffs may visit multiple breeding sites. The number of days that a male is present at a lek varies considerably, at least among independents and satellites, and many males stay at a given lek only for one or several days (fig. 4 in [17]). This short tenure at individual breeding sites is similar to that observed in male pectoral sandpipers [5], and appears to be related to their local success. Independent males that are able to defend a territory, and satellites that are tolerated on the lek by independents, tend to remain at a given breeding site for longer [17]. Independents that do not secure a territory, and satellites that are not accepted on the lek by independents, tend to leave [17]. Faeder males are rare and easily escape detection because of their female-like plumage. Hence, little is known about their breeding site attachment, although van Rhijn remarked that naked-nape males (presumably referring to what is now known to be the distinct faeder morph) ‘rarely visit a lek for more than a few days’ [9, p. 49]. No tracking studies have been performed on this species, however, and it remains unknown what males do after leaving a particular breeding site.

We studied the movements of male ruffs during the breeding season using satellite telemetry data from individuals caught at a stop-over site in spring. Our first aim was to provide a comprehensive description of the movements and sampling of breeding sites of male ruffs throughout their breeding range. Such a description can inform models on dispersal behaviour that require knowledge of the spatial and temporal scale of movements [18]. Our second aim was to compare the movements of the three male morphs. Although it is difficult to make *a priori* predictions, the three morphs have distinct mating strategies at a given breeding site and one can thus expect that they also differ in breeding site sampling behaviour. For example, male independents and satellites that are unable to secure access to a territory on a given lek may benefit from moving to a different site, assuming that they can be competitive and mate successfully elsewhere. By contrast, faeders steal or sneak copulations, so their success depends on the presence of territory owners on the lek and not on their local competitive ability. Consequently, faeders may benefit little from moving between sites. Sampling behaviour of independents and satellites may not differ, given that both groups of males show similar variation in lek attachment, with a similar distribution of local lek tenure [17]. However, unlike resident independent males, satellite males can be resident on multiple (nearby) leks [17], and may therefore move more than independents, also on a larger scale.

## 2. Methods

### (a) Field procedures

We caught 99 male ruffs in a 400 km^2^ area (centre: 52°58’ N, 05°24’ E) in Friesland, The Netherlands, between 14 March and 22 April 2015, using a traditional method with decoys and a clap net (‘wilsternetting’ [19]). The catching area is located at the western limit of the ruffs’ global distribution and is an important stop-over site during their spring migration [20–22]. Individuals use the area to replenish resources and to moult (at least partly) into their breeding plumage before continuing their journey [21–23]. Of the 99 tagged ruffs, 84 were independents, 13 were satellites and two were faeders. The morph of each male was determined using a set of diagnostic molecular markers (single nucleotide polymorphisms [13]), which corroborated our assessment in the field. We also determined age class (yearling/second-year versus adult/after-second year) based on plumage characteristics [24]. Out of 83 independents (for one individual age class could not be determined), 74 were adults and nine were juveniles. Only one among the 13 satellites was a juvenile, and both faeders were adults.

Each individual received a white leg flag and a unique combination of four colour bands. We also equipped each individual with a 5 g Solar Argos PTT−100 (Microwave Telemetry Inc., Columbia, MD, USA) satellite transmitter using a wing harness made out of elastane. The mean body mass of the tagged individuals was 177 g (range: 131–207 g), so the tag added on average 2.8% to the body mass (range: 2.4–3.8%). Of the 99 tagged ruffs, 82 individuals were tracked at least until the start of the breeding season (see §2b(ii) ‘Definition of the breeding season’ below) and moved away from the capture site (68 independents, 12 satellites, two faeders). Sixteen tags stopped sending data early owing to either tag or harness failure, or to bird mortality. One tagged individual stayed in the capture area during the breeding season and later transmitted briefly from an area approximately 70 km away from the capture site. This individual could have reproduced in Friesland (the area used to be a breeding area, but nowadays breeding is scarce [25]) , but we excluded it because of the gaps in data transmission. Of those 82 individuals, 65 (79%) were followed throughout the entire breeding season, for a period of 55.2 ± 10.9 days (mean ± s.d.). An additional four individuals were tracked for more than 40 days, and these were (conservatively) included in the sample of individuals with ‘complete’ monitoring during the breeding season. The remaining 13 males were followed for 19.8 ± 9.6 days. Presumably, their tag fell off because the harness material (elastane) was not durable. This is supported by the observation of eight tagged individuals that were observed in Friesland in subsequent years without the tag. It is also possible that tags stopped working because of technical issues (e.g. loss of battery power), but based on our experience with the same tag type on other birds using a more durable harness material, tag failure seems unlikely.

### (b) Spatial analysis

#### (i) Breeding range

We obtained the breeding range of ruffs from BirdLife International and the Handbook of the Birds of the World [26]. To identify potential breeding habitats, we compared the known breeding range with the distribution of terrestrial ecoregions worldwide, as documented by Olson *et al*. [27]. Any terrestrial ecoregions that overlapped with the known breeding range were considered as potentially suitable breeding habitats (see the electronic supplementary material, figure S1).

#### (ii) Definition of the breeding season

We included only data from the period during which fertile females were presumably present in at least part of the breeding range, which we refer to as the ‘breeding season’. We defined the breeding season conservatively as the interval between the first recorded nest initiation date (first egg date) −7 days and the last recorded nest initiation date. We obtained nest initiation dates from different locations throughout the species’ breeding range [28,29]. Based on this information, the breeding season of the ruff lasts from 13 April until 1 July. Following this definition of the breeding season, all residency sites of male ruffs (see below) were located within the known breeding range of the species or in areas with suitable breeding habitat.

### (c) Data processing

#### (i) Argos data pre-processing

All spatial transformations and operations were done using the R package sf [30]. All points and polygons were transformed to Lambert azimuthal equal-area projection with a longitude centred at 45°. Of all Argos locations, 22.1% had an error radius of 1500 m or less (classes 1, 2 and 3), 18.5% had an error radius of more than 1500 m (class 0), and 59.4% had no associated error radius estimation (classes A and B). The error radius information was subsequently used in modelling the tracks using continuous-time correlated random walk (CTCRW) models (see §2c(iii) ‘Track estimation’). We filtered Argos location data to remove erroneous locations using a speed filter with a maximum ground speed of 150 km h^−1^ [5]. Overall, 4.8% of the data points were removed by this filter.

#### (ii) Residency area identification

Residency areas were identified using a density-based spatial clustering of applications with noise (DBSCAN) algorithm implemented in the R package clusterTrack [31], which is an extension of the package dbscan [32]. As with the dbscan algorithm, clusterTrack starts by detecting spatial clusters of points with different sizes and shapes using two input parameters: the minimum number of points (MinPts) needed to form a cluster and the radius (ε) that defines the neighbourhood of a point. The identification of a cluster starts with an arbitrary point and defines its *ε*-based neighbourhood. This neighbourhood includes all other points within a distance of *ε* from the target point. If the number of points in the neighbourhood is greater than or equal to MinPts, a cluster is formed. This process is repeated for each unvisited point until all points are classified as either cluster points or non-cluster points.

Dbscan does not consider time, so it may classify points as part of the same cluster even if the points form temporally distinct clusters (i.e. the individual left the area of the cluster and returned later to the same general location). To mitigate this potential issue, clusterTrack calculates a temporal contiguity index between points of the same cluster using network analysis [33]. The method entails generating a network graph for each cluster, with the edges in the graph indicating the relative temporal contiguity values. These values determine the ranking of the edges, with closer points in time having a lower rank. A maxLag temporal parameter is used to subset the graph, retaining only edges with a rank value smaller than or equal to the maxLag. Using this technique one putative cluster is split into two or more temporally distinct clusters. To ensure the robustness of the new clusters, clusterTrack re-runs the dbscan algorithm on each newly identified cluster using the initial values for MinPts and *ε* parameters.

We started with the initial values for MinPts = 8 and *ε* = 6.6 km as in [5] and with an arbitrary maxLag value of 10. A visual inspection of the clusters showed that those initial parameter values misclassified some of the track points as clusters. Most final values of *ε* were set to 5 km (*n* = 61) or to 6 km (*n* = 15). Six individuals had either smaller *ε* values: 4 km (*n* = 3) or larger *ε* values: 7 km (*n* = 1), or 8 km (*n* = 2). The final maxLag value was set to 16 for most males (*n* = 73), while nine individuals had larger maxLag value (median = 18, range = 17–25).

By using the earliest and latest location points within a cluster, we estimated the arrival and departure date and time for each residency area, and subsequently calculated tenure values for each male and residency area as the time difference (days) between the arrival and departure.

#### (iii) Track estimation

Tracks were estimated using a CTCRW separately for each individual using the R package aniMotum (formerly known as foieGras) [34]. The CTCRW model consisted of a movement model and an error model incorporating Argos errors. The model was used to create movement tracks based on predicted maximum likelihood locations every 15 min. These tracks were intersected with residency area polygons (computed as convex hulls), discarding values inside each polygon.

### (d) Behavioural variables

Using the residency area clusters and the tracks, the following parameters were obtained for each male: number of sites visited, number of sites visited per week, total distance travelled (km), distance travelled between sites (km), time spent travelling (% of total time), average tenure (days), maximum tenure (days), probability of site revisit (a site is considered re-visited if the proportion of the convex hulls between two sites is more than 50%, mean ± s.d.: 79% ± 17%), probability of occurrence of the longest tenure (a binary variable that indicates whether a site has the longest tenure or not) and bearing variance (computed as angular variance of the direction between successive sites). If male movements are goal-oriented, we expect differences in bearing variance before and after an individual reaches the site with the longest tenure (i.e. the presumed ‘goal’).

### (e) Statistical analyses

Generalized linear mixed models were fitted using the R package glmmTMB [35]. To test for statistical differences between groups we ran pairwise *post hoc* comparisons using the *emmeans* R package [36]. All *p*-values for the post hoc comparisons were adjusted using the Tukey method for multiple comparisons [36].

To test for differences in breeding site sampling behaviour between male morphs, we performed different general linear (mixed) models with the number of sites visited, total distance travelled, distance travelled between sites, time spent travelling, average tenure or probability of site revisit as response variables. Morph (i.e. independent, satellite or faeder) was included as the explanatory variable and male identity was included as a random intercept where applicable.

An additional question is whether males migrate with a particular ‘final breeding destination’ as the aim. Previous studies on birds have shown that the pre-breeding (spring) migration is generally faster, with higher flight speeds and shorter stop-over durations, than the post-breeding (autumn) migration [37]. If the movement patterns described in this study are typical migratory movements rather than breeding site sampling, male ruffs should be moving towards a ‘final breeding destination’, where they stay during the mating season before eventually moving away (post-breeding). In that case, their movement patterns towards this breeding site should differ from those away from that site. Specifically, if male ruffs are ‘goal-oriented’, we predict that (i) they fly to the site with the longest tenure during the breeding season in a more direct manner (with lower bearing variance), and (ii) that they have shorter tenures before they reach this site than after they leave it again. By contrast, if males do not have a final breeding destination but visit different breeding sites opportunistically, we expect that their behaviour before reaching the site where they had the longest tenure will not differ from their behaviour after leaving this site. To test these predictions, we compared the bearing variance and the mean tenure before and after an individual visited the site with the longest tenure using general linear mixed models, with bearing variance or mean tenure as the response variable, period (i.e. before or after staying at the site with longest tenure) as the explanatory variable, and male identity as a random intercept.

To test for differences between age classes in the four main variables describing sampling behaviour among independent males, we performed different general linear models with the number of sites visited, total distance travelled, average tenure or maximum tenure as the response variable. Age class (i.e. yearling or adult) was included as the explanatory variable.

For the statistical analyses involving the variables (i) number of residency areas visited per week (i.e. site visitation rate), (ii) distance between consecutive residency areas, (iii) proportion of time spent flying, and (iv) average tenure we included all individuals (68 independents, 12 satellites and two faeders) that moved away from the capture site during the breeding season. For the analyses involving the variables (v) total number of residency areas, (vi) total distance travelled, (vii) maximum tenure, (viii) probability of occurrence of the longest tenure, (ix) probability of a residency area revisited, (x) bearing variance before and after reaching the site of longest tenure, and (xi) mean tenure before and after reaching the site of longest tenure we included all 69 individuals that were tracked until the end of the breeding season (see above) after leaving the capture site (58 independents, nine satellites and two faeders).

## 3. Results

**Figure 1.**
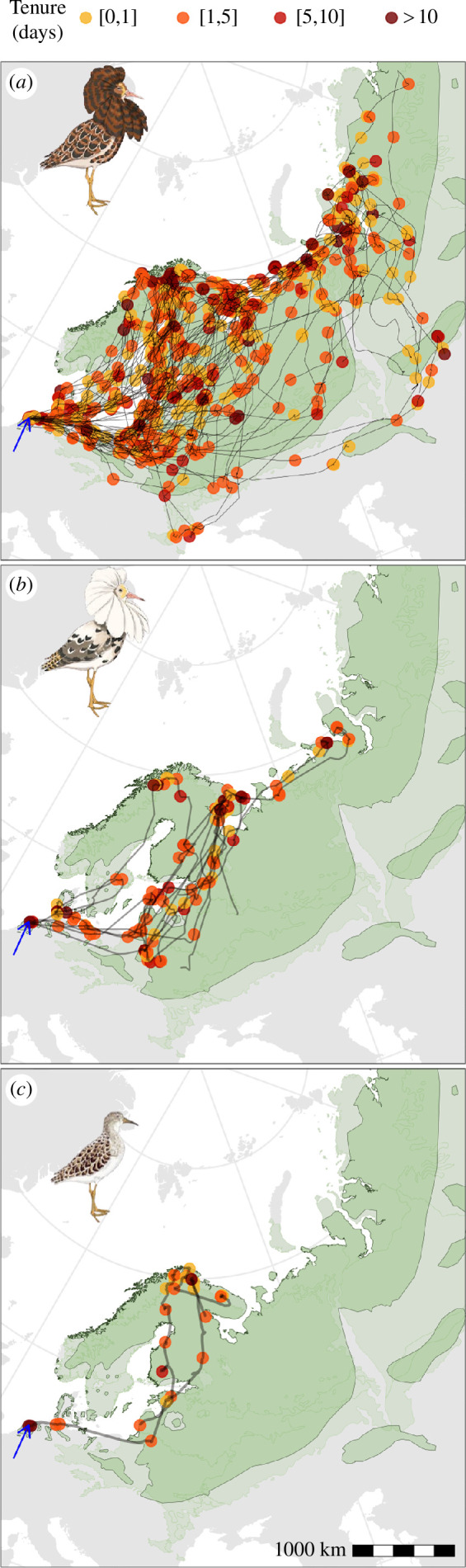
Sites visited by (*a*) independent (*n* = 58), (*b*) satellite (*n* = 9) and (*c*) faeder (*n* = 2) male ruffs caught and tagged in the Netherlands (Friesland). Only movements made during the breeding season are shown (see §2 for definition). Each dot shows the mid-point of a residency site. The line connecting the sites represents the estimated movement path. The colour of a dot indicates tenure, which is defined as the duration of occupation of a given site (calculated as the duration between the initial and final recording at that location, §2). The dark green area indicates the species’ known breeding range; the light green area shows the area outside the known breeding range with suitable breeding habitat. The blue arrow points to the capture site. Map projection is polar Lambert azimuthal equal-area. Details on all individual birds can be found at http://ornithology.bi.mpg.de/ESM/Kempenaers_et_al_2024. Ruff illustrations: Maayan Harel, Maayan Visuals.

**Figure 2.**
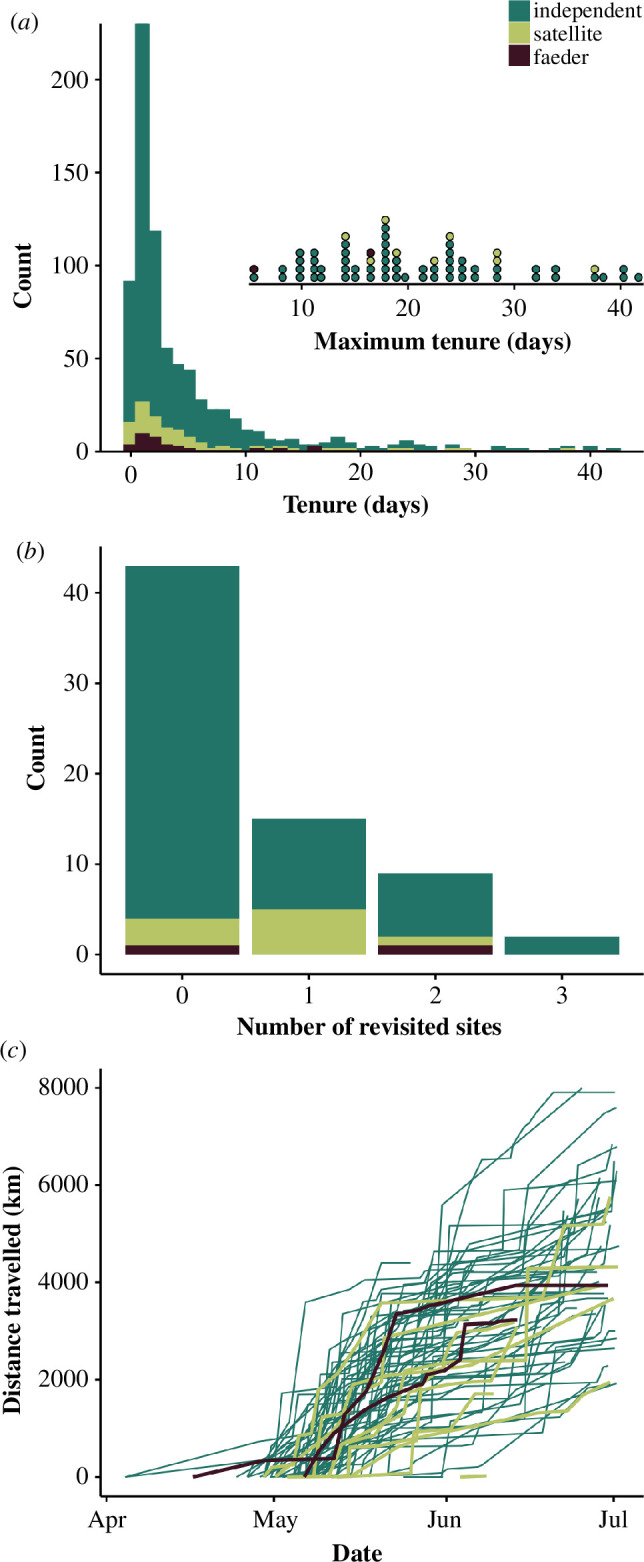
Characteristics of breeding site sampling of the three ruff morphs (independents: *n* = 58, satellites: *n* = 9, faeders: *n* = 2; shown in different colours). (A) Frequency distributions of tenure at each site. The inset shows the longest recorded tenure of each male. (B) The total number of individuals that revisited zero to three residency areas. Shown are stacked bars. (C) The cumulative distance travelled during the breeding season. Lines connect successive points whereby the first point corresponds to the arrival date at the first residency area after leaving the capture site.

**Figure 3.**
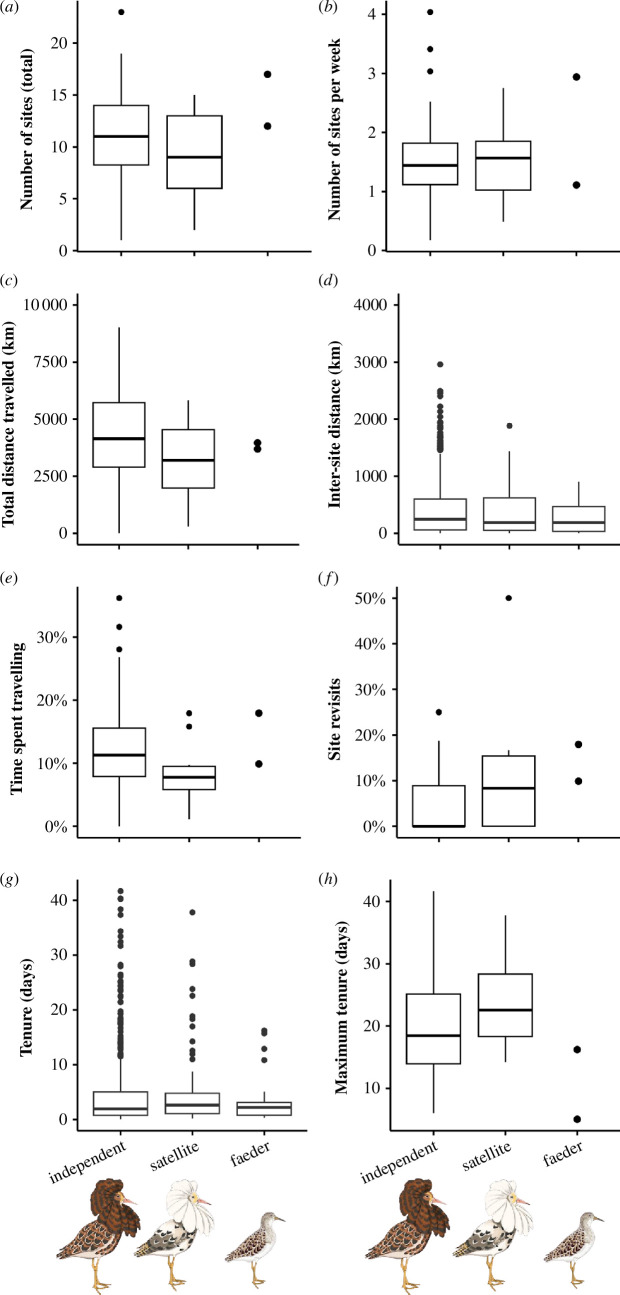
Comparison of descriptors of breeding site sampling behaviour of males of the three ruff morphs. (A) The total number of residency areas (sites) visited by each individual during the breeding season. (B) The number of residency areas visited per week. (C) The total distance travelled during the breeding season (in km). (D) The distance between consecutive residency areas (in km). (E) The proportion of the total time spent flying between residency areas. (F) The proportion of residency areas that were revisited by the same individual in the same year. (G) The tenure at each residency area, i.e. the duration of occupation of a given site, calculated as the duration between the initial and final recording at that location (in days). (H) The longest duration an individual spent at any site within a given season (maximum tenure, in days). Shown are boxplots with median (centre line), 25−75th percentile (limits), minimum and maximum values without outliers (whiskers) and outliers (dots). For faeders, the raw data are shown because only two datapoints were available (except for panels (D) and (G)). Ruff illustrations: Maayan Harel, Maayan Visuals.

Ruff males that were caught in Friesland during spring migration at the western limit of their global distribution proceeded to move across up to 60% of their longitudinal breeding range over the course of the breeding season, with typically only brief stops (figures 1 and 2*a*). In doing so, they visited a median of 11 different potential breeding sites (range: 1–23, figure 3*a*) and travelled a median distance of 4435 km (range: 0–9028 km, figure 3*c*), with a median distance between sequentially visited sites of 210 km (range: 1.5–2747 km, figure 3*d*). Males spent a median of 9.6% of their total time within the breeding range moving between sites (range: 0–28%, figure 3*e*), and the median amount of time spent at a particular site (tenure) was 2 days (range: 0.02–41.7 days, figure 3*g*). Only 4.8% of the sites were revisited (figures 2*b* and 3*f*). For male ruffs that could be tracked during the entire breeding season ( > 40 days), the maximum longest tenure was 41.7 days and the minimum longest tenure was 5.0 days; figures 2*a* and 3*h*).

Satellites, independents and faeders made similar movements during the breeding season (figure 1). The three morphs did not differ in the number of sites visited (figure 3*a*,*b*; electronic supplementary material, tables S1 and S2), the total distance moved within the breeding area (figure 3*c*; electronic supplementary material, table S3), the distance moved between sequentially visited sites (figure 3*d*; electronic supplementary material, table S4), the proportion of time travelling between sites (figure 3*e*; electronic supplementary material, table S5), the average amount of time spent at a particular site (figure 3*g*; electronic supplementary material, table S6), the probability of revisiting a site (figure 3*f*; electronic supplementary material, table S7), or the longest tenure at any given site (figures 2*a* and 3*h*; electronic supplementary material, table S8).

**Figure 4.**
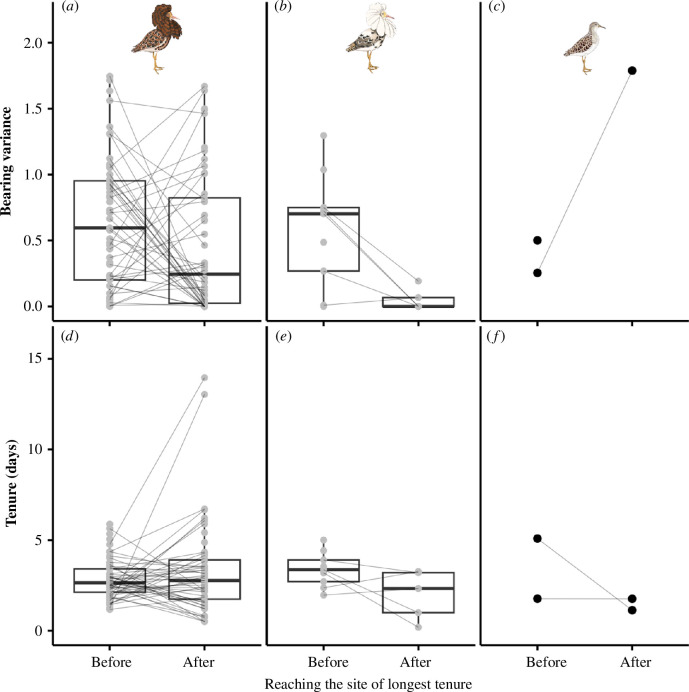
Comparison of movement behaviour of male ruffs of the three genetic morphs (independents: *n* = 58, satellites: *n* = 9, faeders: *n* = 2) before and after the individual arrived at the residency area with the longest tenure (i.e. the potential target area, under the hypothesis that birds are goal-oriented). (*A–*C) Bearing variance, calculated as the angular variance of the direction between successive sites. (*D–*F) Average tenure at all sites visited before or after the visit to the site with longest tenure. Ruff illustrations: Maayan Harel, Maayan Visuals.

Movements were not limited to a particular part of the season (figure 2*c*). Among males that were recorded during the entire breeding season (after leaving the capture site), the bearing variance and the average duration of tenure at each residency area did not differ between the period before and after visiting the area with the longest tenure (figure 4; electronic supplementary material, tables S9 and S10). The residency area with the longest tenure could be any of the visited areas, including the first or the last one (electronic supplementary material, figure S2).

In independents, age class did not explain variation in sampling behaviour: yearling and adult males did not differ significantly in the number of sites they visited, in total distance travelled, or in average or maximum tenure (electronic supplementary material, tables S11–S14). However, these results remain inconclusive owing to the small number of yearlings tracked (*n* = 5).

## 4. Discussion

Ruff males caught at a stop-over site at the western limit of their range during spring migration proceeded to travel across maximally approximately 60% of their longitudinal breeding range. The visited range coincided with the suggested flyway towards the Fenno-Scandinavian, the European Russian Arctic, and the Western and Central Siberian regions (see fig. 1 in [22]). Males visited up to 23 different sites and travelled up to 9029 km during the breeding season (figures 1 and 3), with no detectable seasonal pattern (figure 2*c*) and no evidence for a ‘target destination’, i.e. a particular area that was the intended goal of the movement (figure 4; electronic supplementary material, figure S2). Note that our method underestimated the true level of sampling of potential breeding sites or leks, because (i) not all individuals were tracked until the end of the breeding season, (ii) local sampling of different leks is not included (owing to limitations on the spatial accuracy of the data), and (iii) short visits to a lekking area or areas visited while few data were transmitted are missed, because at least eight locations are needed for a cluster to be defined as a residency area (see §2).

The breeding site sampling behaviour of the ruff is similar to that described for another migratory species also characterized by strong male–male competition and female-only care. In the polygynous pectoral sandpiper, most males also travelled across large parts of their breeding range, visited multiple sites and covered thousands of kilometres during a single breeding season [5]. We suggest that large-scale breeding site sampling is not unique to these two closely related species, but may be a common strategy among males of migratory species with high male–male competition and female-only care in which local mating opportunities vary in time and space. For example, a recent study on the lekking great snipe *Gallinago media* showed that 75% of males moved between leks, and that males visited up to nine different leks within a breeding season, with the distance between these leks varying between less than 50 km and 253 km [38]. Because data needed to be locally downloaded, only males that returned to a studied lek were recorded, so the true level and extent of sampling in this species is probably higher.

If the movements of male ruffs between breeding sites are mainly related to an individual’s relative competitive ability at a lek, we would predict that less competitive independent and satellite males, e.g. young, inexperienced individuals or individuals with a lower competitive ability, show the highest degree of breeding site sampling or are the only ones that show this behaviour. This prediction is based on the assumption that males can quickly assess their probability of local mating success, as may be the case if aggressive interactions lead to fast establishment of dominance relationships [39]. Satellites may show less breeding site sampling if they have more opportunities to mate locally, which may be the case because—in contrast to independents—some are attached to multiple nearby leks [17]. The opposite prediction can also be made, based on previous studies suggesting that satellites are the more mobile morph [9,11], or based on the assumption that the cost of movement would be higher for less experienced or lower quality males.

In contrast to these expectations, we found no differences in the within-breeding season movements between yearling and older independents (electronic supplementary material, tables S11–S14), or between the three morphs. Independents, satellites and faeders travelled similar distances, visited a similar number of sites, and remained at each site for a similar amount of time (figure 3). Although these findings are preliminary, given the low sample size of yearlings and of faeders, they at least suggest that breeding site sampling may be caused by changes in the local availability of fertile females, which affects the reproductive opportunities at a particular site—and thus the benefits of staying versus leaving—similarly for all individuals. Vervoort & Kempenaers [17] already observed that independents and satellites did not differ overall in local lek tenure, and that many individuals only had short tenures at a given lek. However, it was unknown where these individuals went, and one could also have assumed that they behaved as ‘failed breeders’, for example foraging along the coast and starting their post-breeding migration. Our results clearly show that this is not the case.

We hypothesize that male movement decisions depend on local mating opportunities, but information on the spatio-temporal distribution of fertile females is often unavailable (see fig. 3*b* in [5]). We therefore need data on the number of fertile females that visit a lek or a local breeding area over the season (see fig. 1*e*,*f* in [40]). We expect males to sample more sites if relatively few females visit a given lek and local female fertility is highly synchronous. Because male movements depend on where the females are, we also need more information about female movements before and during the breeding season. Females may also sample many potential breeding areas before they settle, for example to assess the local ‘qualities’ for nesting (e.g. food availability, predation risk; see [41]), or they may simply return to the area where they bred the previous year. Furthermore, we need more information about female behaviour after (early) nest failure. Do females move locally or over longer distances to renest? A recent study on the American woodcock *Scolopax minor* showed that most females nested multiple times and moved on average 800 km northwards between their first and second nesting attempt [42]. This study suggests that females can also move over large areas within a breeding season. The authors suggest that the use of ephemeral breeding habitat and the low cost of movement may be key factors explaining this ‘itinerant breeding’ behaviour.

A parallel can be drawn between breeding site sampling and dispersal (*sensu* [43]). Theory suggests that male and female movement strategies are driven by multiple factors, including mating/breeding opportunities, the competitive conditions at a given site, the time or energy an individual needs to invest to assess the probability of successful mating/breeding at a site (e.g. the local sex ratio, predation risk), and the costs of dispersing [18,43,44]. One can therefore expect variation among individuals and among species in the frequency of decisions to leave a site, as well as in the distance and direction of movement. Two types of factors may influence the occurrence or extent of breeding site sampling of a species or population: (i) environmental heterogeneity (e.g. unpredictability in the location or timing of availability of suitable breeding habitat and hence of mating opportunities, variation in local sex ratios); and (ii) species characteristics (e.g. migration distance, mating system). We describe four expectations about the occurrence of breeding site sampling.

First, species with a breeding range encompassing a wider range of breeding schedules among females may perform more sampling behaviour, because high between-site temporal variation in the availability of fertile females increases sequential mating opportunities for males.

Second, breeding-range sampling may be expected to be more common in species with a higher reproductive skew. In particular, if one or a few dominant males locally restrict the ability of other males to gain access to females, such successful males should stay while unsuccessful males would benefit from moving, assuming that they can be competitive and mate successfully elsewhere.

Third, we suggest that in sex-role reversed species, where offspring care is performed by males and females compete for access to males [45], females may also perform breeding site sampling similar to that shown in males of polygynous species. However, females may be more constrained because they need time to lay their clutch. Thus, the likelihood that breeding site sampling evolves in females may depend on the availability of males as carers, but also on the total length of the breeding season and on spatial variation in the optimal timing of breeding.

Fourth, we expect breeding site sampling to be more pronounced in species with longer migration distances, because they already have the adaptations for long flights, which may reduce the costs of such sampling movements. For example, in the wild turkey *Meleagris gallopavo*, a non-migratory polygynous species with female-only care, males did not move large distances within a season, although they did increase the spatial scale of their daily movements during the mating period, presumably to maximize encounters with receptive females [46].

Large-scale breeding range sampling has important implications for population ecology and for evolutionary processes. First, seasonal changes in the presence of males (or females) can influence the sex ratio of local breeding populations, which may affect mating strategies [47,48]. Second, the reproduction of individuals across a large part of the species’ breeding range even within a single season results in high gene flow between breeding areas, leading to a panmictic population with little genetic differentiation, and hence little opportunity for local adaptation and the evolution of subspecies [5,49,50]. Third, large-scale movements can have consequences for the degree of reproductive skew and the strength of sexual selection [51]. Male reproductive success is typically assessed locally at a particular breeding site. However, if individuals can successfully reproduce at multiple sites, variance in male reproductive success may be considerably higher than estimated based on local field studies. This would be especially true in ruffs, if males can sire offspring during short tenures and progressively move further north following the advance of spring, as suggested by van Rhijn [9]. Conversely, if males that are unsuccessful at a given lek sample other leks until they find a mating opportunity (as suggested by [41]), among-male variance in reproductive success may be substantially lower than estimated based on measures at a given study site.

In conclusion, we show that male ruffs perform large-scale movements throughout a substantial part of their breeding range during a single breeding season. Their annual cycles are thus not characterized by a clear distinction between a migratory period with much movement and a breeding period of residency. Rather, ruff males show a pattern of what looks like nomadic movements, that is, movements that are irregular in space and time. Our study leads to a general question: what are the conditions that are required for male nomadic behaviour during the breeding season to evolve? To answer this question, we need more empirical data as well as modelling approaches. Although Shaw & Kokko [18] and Li & Kokko [43] lay out the broad conditions under which dispersal behaviour (or ‘restlessness’) of both sexes can evolve, formal theory on the link between the mating system and movement patterns is largely lacking.

Large-scale breeding site sampling may be a common strategy in males of species with female-only care and high temporal and spatial variability in the availability of fertile females. This leads to several follow-up questions. First, do these species have physiological adaptations to cope with extreme endurance and to enable such continued long-distance flights? So far, studies on pectoral sandpipers have shown that during the breeding season males can function with very little sleep [6] and sustain unusually high haematocrit levels, suggesting that they maintain high aerobic capacity equivalent to that during migration [52]. Second, how do males decide to stay or to leave, and if they leave, how do they decide where to go next? Third, and following from the previous question, do individuals fly by themselves or in groups with other males or with females? Fourth, how does this behaviour change between seasons and over the lifetime of an individual? Fifth, does this nomadic behaviour also occur in the non-breeding season, that is, is it part of a ‘nomadic syndrome’? Observations in spring of high heterogeneity in stopover duration among individuals and low between-year repeatability of individual stopover behaviour [23] suggests that this might be the case. However, data from the non-breeding season are needed, because individuals could still be highly faithful to a wintering site and become flexible during migration, related to variation in environmental conditions. Finally, we need to ‘cherchez la femme’, that is, to study female movements before and during the breeding season, because male movements might be driven largely by the behaviour of females.

## Data Availability

All data and code can be found here: [53]. The tracks and residency areas of all individual birds can be found at http://ornithology.bi.mpg.de/ESM/Kempenaers_et_al_2024. Supplementary material is available online [54].
